# Slowing down or returning to normal? Life expectancy improvements in Britain compared to five large European countries before the COVID-19 pandemic *and* Monkeypox: a review of the 2022 outbreak

**DOI:** 10.1093/bmb/ldad005

**Published:** 2023-04-05

These important subjects have been chosen to have free online access. The British Medical Bulletin website also has a section to celebrate its amazing archive (see https://academic.oup.com/bmb/pages/from-the-archives) and details of the Nobel Prize-winners who wrote for the Bulletin and went on to win the accolade. It also has information on which reviews have been most widely cited and information about the OUP blog, which often has input from the Bulletin authors and editors.

The first free online access review is: **Slowing down or returning to normal? Life expectancy improvements in Britain compared to five large European countries before the COVID-19 pandemic**  *by Minton, Hiam, Dorling and McKee from the University of Glasgow, University of Oxford and the London School of Tropical Medicine and Hygiene, UK.* They say life expectancy is an important summary measure of population health. In the absence of a significant event like war or disease outbreak, trends should and historically have, increased over time, albeit with some fluctuations. Life expectancy improvements in Great Britain have stalled in recent years, and a similar stalling was seen in other high-income countries during the mid-2010s. The significance and causes of the slowdown in improvement in life expectancy in Britain are disputed. Other measures, such as lifespan disparity complement it in understanding changing trends. Whilst annual fluctuations in life expectancy are expected, continued stalls should raise concern. The three British nations examined were the only ones amongst these European countries to experience stalling of life expectancy gains in both sexes. Whilst Britain is making less progress in health than similar countries, more research is needed to explain why.

The second free-to-view review is entitled: **Monkeypox: a review of the 2022 outbreak**  *by Lim, Whitehorn and Rivett from the Cambridge University Hospitals NHS Foundation Trust and Public Health Laboratory, Cambridge, UK.* They say that In May 2022, the World Health Organization declared a multi-country monkeypox outbreak following cases reported from 12 member states which were not endemic for monkeypox virus. There are variations in clinical presentations seen in the current outbreak, which has not been seen in prior outbreaks. More research is needed to investigate the reasons for these differences. The higher number of human immunodeficiency virus (HIV)-positive patients in the current outbreak has allowed a better description of the disease in patients co-infected with HIV and monkeypox. The absence of more severe symptoms in HIV-positive patients in the current outbreak could possibly be because most of these patients had well-controlled HIV. Current treatment and vaccination options have been extrapolated from studies of other Orthopox viruses. There remains a need for more data on the safety and efficacy of these options in the context of monkeypox infections.

In the rest of this volume the third review is titled: **Return to sport or work following surgical management of scapholunate ligament (SLL) injury**  *by Liew, Dingle. Semple and Rust from the University of Edinburgh and the University of Manchester, UK.* They say that their systematic review aims to compare the rate and time to return to sport or work following surgical interventions for isolated SLL injury. Fourteen papers, including six different surgical interventions, met the criteria for final analysis. All surgical techniques demonstrated acceptable rates of return to work or sport (>80%). The optimal surgical intervention for isolated SLL injury remains undetermined due to heterogeneity and limited sample sizes of published studies. This systematic review has provided clarification on the available literature on treatment modalities for isolated SLL injuries in the absence of osteoarthritis (OA). Prospective, randomized, primary studies are needed to establish optimal treatment for acute isolated SLL injuries.

The fourth review is entitled: **Femoroacetabular impingement syndrome negatively affects the range of motion of the affected hip**  *by Albertoni, Bargen, Hoxha, Munari, Maffulli and Castellini from the University of Genoa, Scientificao Ortopedico Galeazzi, Insituto di Sanito Pubblica Italy; the Barts and London School of Medicine, London, UK.* They say it is unclear whether femoroacetabular impingement syndrome (FAIS) affect hip range of motion (ROM).

A total of 17 studies were included. Comparison of FAIS patients versus healthy controls showed that hip ROM was clinically and statistically reduced in FAIS for internal rotation, hip flexion; adduction and flexion-abduction and external rotation ranging from low to high certainty of evidence. Comparison of FAIS versus healthy controls showed no statistically significant differences in any direction of movement, albeit with uncertainty of evidence. Hip ROM may be reduced in all directions except extension in FAIS compared to controls.

The fifth review is entitled: **Cancer survivors and adverse work outcomes**  *by de Boer, de Wind, Coenen, van Ommen, Greidanus, Zegers, Duijts and Tamminga from the University of Amsterdam, Amsterdam Public Health, Amsterdam Cancer Centre, Amsterdam; Movement Sciences and the Netherlands Comprehensive Cancer Organization, Utrecht, the Netherlands.* They say that the number of cancer survivors of working age is rising. A range of factors is associated with adverse work outcomes such as prolonged sick leave, delayed return to work, disability pension and unemployment in cancer survivors. They also include cancer type and treatment, fatigue, cognitive functioning, work factors and elements of health care systems. Effective supportive interventions encompass physical and multicomponent interventions. The role of behaviour determinants and of legislative and insurance systems, is unclear. The optimal timing of delivering supportive interventions is uncertain. Further focus on vulnerable groups, including, specific cancer types and those with lower income, lower educational level and in precarious employment, is needed.

The sixth review is entitled: **Knee osteoarthritis, joint laxity and patient recorded outcome measures (PROM)s following conservative management versus surgical reconstruction for anterior cruciate ligament (ACL) rupture**  *by Migliori, Oliva, Torsiello, Eschweiler, Hildebrand and Maffulli from the University of Salerno, Italy; University of Aachen, Germany and Queen Mary University of London, UK.* They say that patients who rupture their ACL can be managed conservatively or undergo reconstruction surgery. Several studies published by July 2022 compare surgical and conservative management following ACL rupture. The latest evidence suggests that surgical management may expose patients to an increased risk of early onset knee OA. After the initial trauma, surgical reconstruction may produce more damage to the intra-articular structures compared to conservative management. The present study compared surgical reconstruction versus conservative management for primary ACL ruptures in terms of joint laxity, patient reported outcome measures (PROMs), and rate of OA. ACL reconstruction provides significant improvement in joint laxity compared to conservative management, but is associated with a significantly greater rate of knee OA, despite similar results at PROMs assessment.

The seventh review is entitled: **Methods of assessing value for money of UK-based early childhood public health interventions**  *by Richardson, Murphy, Hinde, Fulbright and Padgett from the University of York.* They say that economic evaluation has an important role to play in the demonstration of value for money of early childhood public health interventions, however, concerns have been raised regarding their consistent application and relevance to commissioners. This systematic review of the literature aims to collate the breadth of the existing economic evaluation evidence of these interventions and to identify the approaches adopted in the assessment of value. This review considered inconsistencies across methodological approaches used to demonstrate value for money. Future resource allocation decisions regarding early childhood public health interventions may benefit from consistency in the evaluative frameworks and health outcomes captured, as well as consistency in approaches to incorporating non-health costs and outcomes; incorporating equity concerns; and the use of appropriate time horizon.

The eighth review is entitled: **Children and bioethics: Clarifying consent and assent in medical and research settings**  *by Spriggs from the University of Melbourne, Australia.* She says that the concept of consent in the paediatric setting is complex and confusing. Clinicians and researchers want to know whose consent they should obtain, when a child can provide independent consent, and how that is determined. The aim of this article is to establish what produces the justification to proceed with medical or research interventions involving children and the role of consent. It clarifies concepts such as consent, assent, capacity and competence. Engaging with children and involving them in decisions about matters that affect them is a good thing. It examines the role of competence or capacity and the question of when a child can provide sole consent. Flawed assumptions around competence/capacity are common. An account of children’s well-being should accommodate children’s interests during the transition to adulthood.

The ninth review is entitled: **Implementing brief and low intensity psychological interventions for children and young people with internalizing disorders**  *by Roach, Cullinan, Shafran, Heymen and Bennett from the Institute of Child Health and University College, London, UK.* They say that many children fail to receive the mental health treatments they need, despite strong evidence demonstrating efficacy of brief and low intensity psychological interventions. This review identifies the barriers and facilitators to their implementation. Studies identified organizational demands, lack of implementation strategy and stigma as barriers to implementation, and the need for clear training and plans for implementation as facilitators. No standardized implementation outcomes were used across papers so meta-analysis was not possible. Barriers and facilitators have been clearly identified across different settings. Longitudinal studies can identify methods and processes for enhancing long term implementation and considers ways to monitor and evaluate uptake into routine practice.

The tenth review is entitled: **Loneliness—a clinical primer**  *by Lederman from Hong Kong University.* He says loneliness is prevalent worldwide. It is also associated with an increased risk for depression, high blood pressure, cardiovascular disease, stroke and early death. As such, loneliness is a major public health issue. This paper summarizes the salient points clinicians should know and encourages clinicians to assume an active part in the identification, mitigation and prevention of loneliness. Loneliness is a distressful subjective experience, which does not always correlate with social isolation. Identifying loneliness in the clinic may be time consuming and challenging. There is scarce robust evidence to support interventions. More research is needed to further elucidate the health impacts of loneliness as well as to find evidence-based interventions to prevent and mitigate loneliness that could then be implemented by policy-makers and clinicians.

The eleventh revie is entitled: **Micro RNA in meniscal ailments: current concepts**  *by Migliorini, Vecchio, Giorgino, Eschweiler, Hildebrand and Maffulli from the University of Aachen, Germany; the University of Salerno, the Orthopaedic Institute Galeazzi, Milano, Italy; Barts and the London School of Medicine and the University of Keele, UK,* They say Micro ribonucleic acid (miRNAs) are short non-coding RNAs, which act primarily in posttranscriptional gene silencing, and are attracting increasing interest in musculoskeletal conditions. Recently, the potential of miRNAs as biomarkers for diagnosis and treatment of meniscal injuries has been postulated. Evaluation of the role of miRNAs in patients with meniscal tears is still controversial. A systematic review was conducted to investigate the potential of miRNA in the diagnosis and management of meniscal damage. Intra-articular injection of microRNA-210 *in vivo* may represent a potential innovative methodology for the management of meniscal injuries. Characterization of the microRNAs expression in the synovial fluid could lead to the development of better early diagnosis and management strategies for meniscal tears.

**March 2023**

